# Impact of Temporal and Spatial Resolution in Slope-Plant-Atmosphere Interaction Modelling

**DOI:** 10.1007/s10706-026-03733-2

**Published:** 2026-05-19

**Authors:** Maryam Sadat Maddah Sadatieh, Aikaterini Tsiampousi, Athanasios Paschalis

**Affiliations:** 1 Department of Civil and Environmental Engineering, Imperial College London, London, UK; 2 Science and Solutions for a Changing Planet DTP, London, UK; 3 Civil and Environmental Engineering, University of Cyprus, Nicosia, Cyprus

**Keywords:** Soil–plant–atmosphere interaction, Numerical analysis, Stability, Serviceability, Boundary conditions

## Abstract

Soil–Plant-Atmosphere Interaction (SPAI) is an essential factor in slope behaviour, affecting water inflow and outflow, and thereby influencing Pore Water Pressures (PWP), soil strength and stiffness, and slope stability and serviceability. Due to its complexity, SPAI and its effect on slope behaviour are best described by hydro-mechanically coupled numerical analysis, rendering the boundary conditions (BC) used to replicate atmospheric conditions critical. Here, different considerations have been made regarding the temporal and spatial variation of these BCs to assess their effect on slope behaviour. Specifically, daily and monthly atmospheric data were contrasted, dynamic vegetation growth was juxtaposed with static vegetation, and water extraction with depth due to transpiration was compared with a simplified approach where evapotranspiration was modelled to occur from the ground surface. A representative cut slope was considered, and fully coupled hydro-mechanical analyses were conducted under different BCs to study its stability and serviceability. The numerical results highlight which modelling choices significantly influence predicted performance, particularly under climate change, and which can be safely simplified. Guidance is provided for balancing computational efficiency with accuracy in geotechnical design.

## Introduction

Engineered slopes play a critical role in supporting civil infrastructure (Perry et al. 2001, 2003). An important factor in slope behaviour, affecting safety and serviceability, is the amount of water flowing into or out of the slope. This directly affects Pore Water Pressure (PWP) distribution, which in turn affects shear strength and therefore the Factor of Safety (FoS) (Hemmati et al. 2012; Smethurst et al. 2006). Additionally, water inflow/outflow drives soil swelling/shrinking, giving rise to seasonal volume changes which lead to poor serviceability (Tang et al. 2009). Water inflow/outflow is affected by weather conditions and vegetation cover. Several methods exist for modelling Soil–Plant-Atmosphere Interaction (SPAI), including imposing a groundwater head for steady state analysis to represent average winter and summer conditions (e.g., Lollino et al. 2010), adopting summer and winter undrained shear strength profiles (e.g., Vaughan 1994) or summer and winter PWP profiles (e.g., O’Brien et al. 2004), running non-coupled analyses to calculate PWPs and slope stability separately (Ni et al. 2025; Rouainia et al. 2009). More recently, hydrological fluxes were applied as hydraulic BCs in coupled hydro-mechanical analyses (e.g., Tsiampousi et al. 2017, 2024). Earlier studies typically described surface BCs seasonally or monthly, whereas more recent studies have adopted daily fluxes (Jamalinia et al. 2019; Maddah Sadatieh et al. 2025; Ni et al. 2024) or coupled soil-root models with sub-daily fluxes for short representative rainfall patterns (Guo et al. 2024).

A large part of Actual Evapotranspiration (AET) occurs through transpiration, which, in turn, is a function of plant structure, mainly Leaf Area Index (LAI), and the response of plant stomata to weather forcing. LAI is defined as the ratio of leaf area over the ground area beneath it. For example, deciduous vegetation typically has an LAI close to 0 in winter, which then increases by spring and summer, reaching its maximum value. LAI evolves dynamically under the influence of climate, plant type (evergreen or deciduous), and responses to environmental stresses (e.g., leaf shedding due to drought). Ecosystem models can predict the dynamic evolution of LAI, a factor that has often been neglected in geotechnical engineering analysis despite its influence on rainfall interception and evapotranspiration processes (Granier et al. 1999; Yan et al. 2012), and on radiation balances (reflection, transmittance and scattering) (Parker 2020), and ultimately on the net inflow/outflow. Jamalinia et al. (2019) demonstrated a correlation between LAI, infiltration, evapotranspiration and FoS (Jamalinia et al. 2019). To study the effect of vegetation and more specifically LAI on regional hydrological processes and slope stability, Chen et al. (2025) compared dynamic vegetation cover (i.e., with varying LAI) with a static vegetation cover (i.e., with a constant annual LAI and without inter-annual variations) and reported that dynamic vegetation effects should not be neglected (Chen et al. 2025).

Hydraulic BCs have typically been applied as surface BCs to simulate SPAI, although water outflow through transpiration occurs via root water uptake within the root zone. Simplified attempts have been made to represent vegetation-driven water removal at depth, such as implementing internal BCs and prescribing a fixed suction (i.e., tensile PWP) in a region of the soil to mimic plant behaviour (O’Brien et al. 2004). Nyambayo and Potts developed a Root Water Uptake Model (RWUM) that calculates the effect of water removal by roots in different depths of the soil. Transpiration was represented as an internal sink distributed with depth, enabling PWPs to be calculated from plant water extraction rather than being imposed (Nyambayo & Potts 2010). This marked a step forward, particularly as root depth was incorporated in the analysis, and transpiration was distributed with depth. The RWUM of Nyambayo & Potts (2010) translated potential to actual transpiration rates based on the simplistic approach proposed by Feddes et al. (Feddes et al. 1978), which relates soil suction to transpiration reduction factor, thus essentially accounting for water availability indirectly and neglecting other critical plant processes such as dynamic plant growth and the coupling of water, carbon and energy cycles at land surface. As pointed out by Elia et al. (2017), imposing unrealistic BCs without calibration risks producing unrealistic PWPs (Elia et al. 2017). The RWUM was applied by Tsiampousi et al. (2017) to distribute simplified hydrological fluxes with depth (Tsiampousi et al. 2017), within a fully coupled hydro-mechanical analysis of a slope to model vegetation and its contribution explicitly.

In the present study, a physics-based ecohydrological model (T&C) was employed to compute the net inflow/outflow that can be applied in geotechnical numerical analysis. As demonstrated by Maddah Sadatieh et al. (2025), this approach presents the advantage of actual transpiration being computed naturally by T&C, rather than relying on simplified approaches to estimate it. The manner in which T&C outputs can be applied as hydraulic boundary conditions in the geotechnical model was examined comprehensively here, considering three distinct approaches: (1) applying daily vs. monthly flux rates, (2) involving dynamic vs. static vegetation, and (3) assuming surface transpiration vs. transpiration which is distributed with depth. A representative cut slope was considered, and a fully coupled hydro-mechanical analysis was performed in a 2D plane-strain Finite Element slope analysis. PWP profiles, PWP time series near the slope’s toe (0.2 m in depth), FoSs, and vertical displacements from the toe of the slope to the excavation centerline were analysed to assess the effects of temporal and spatial resolution of SPAI BCs on both slope safety and serviceability. PWP profiles were used as they directly influence FoS, and changes in PWP govern the volume change that leads to vertical displacements at the road surface, thereby causing serviceability issues.

## Problem Description

### Geometry and Soil Stratigraphy

**Fig. 1:**
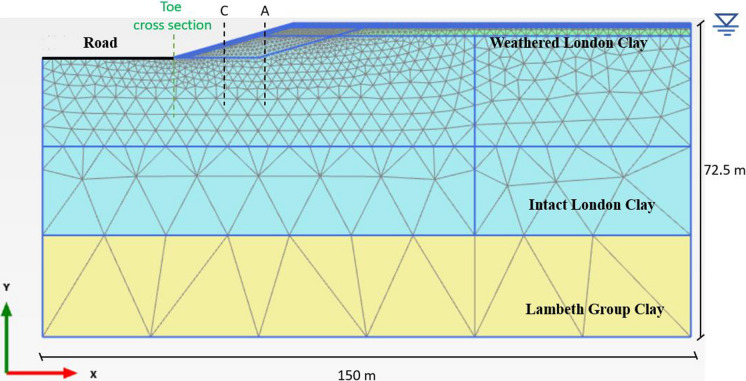
Cut slope’s geometry and stratigraphy modelled in PLAXIS 2D (reproduced from Maddah Sadatieh et al. (2025))

The case study considered here is based on a cut slope located in Newbury, UK. The cut slope is 8 m high and 28 m wide. The soil stratigraphy consists of 3 m of Weathered London Clay (WLC), followed by 46 m of Intact London Clay (ILC) and 23.5 m of Lambeth Group Clay (LGC). The bottom boundary coincides with the upper chalk. The cut slope was excavated in July 1997 and was extensively monitored between 2003 and 2008 (Smethurst et al. 2006, 2012). Figure 1 shows the geometry and stratigraphy of the considered cut slope.

### Mechanical Soil Properties

The analysis was conducted in 2D plane-strain using PLAXIS (version 24.3.0.95) (Sequent 2024). All soil layers were modelled with a Mohr–Coulomb failure criterion with isotropic small-strain stiffness (Taborda et al. 2016). This was implemented in PLAXIS using a User Defined Soil Model (UDSM) (Taborda et al. 2023a, 2023b). The mechanical properties are presented in Table 1. Further to the angle of shearing resistance, the nonlinearity of stiffness with strain level was also of importance, since examining slope serviceability was one of the analysis goals. The same properties were employed by Maddah Sadatieh et al. (2025) and Tsiampousi et al. (2017), and reproduce the stiffness curves reported by O’Brien et al. (Maddah Sadatieh et al. 2025; O’Brien et al. 2004; Tsiampousi et al. 2017). To initialise the stresses, a unit weight of 20 $\mathrm{k}\mathrm{N}/{\mathrm{m}}^{3}$ was chosen for all soil layers. The coefficients of earth pressure at rest are shown in Table 2 (Hight et al. 2007). FoSs were calculated here by running secondary drained analyses at the end of each month using the strength reduction method, i.e. freezing time and calculating FoS for that specific time instance and stress distribution (i.e., the last day of the month) (Potts & Zdravkovic 2012; Tsiampousi et al. 2013).

**Table 1:** Mechanical properties for all soil layers for the geotechnical model

User defined soil model parameters	Values	User defined soil model parameters	Values	User defined soil model parameters	Values
${G}_{ref} [\mathrm{k}\mathrm{N}/{\mathrm{m}}^{2}]$	955	$b [-]$	1.3	${RK}_{min} [-]$	0.079
${K}_{ref} [\mathrm{k}\mathrm{N}/{\mathrm{m}}^{2}]$	1665	${RG}_{min} [-]$	0.05	${K}_{min} [\mathrm{k}\mathrm{N}/{\mathrm{m}}^{2}]$	3000
${p}_{ref} [\mathrm{k}\mathrm{N}/{\mathrm{m}}^{2}]$	1	${G}_{min} [\mathrm{k}\mathrm{N}/{\mathrm{m}}^{2}]$	2000	$\varphi [^\circ ]$	23
${m}_{G} [-]$	0.7	${r}_{0} [-]$	0.3E-3	$c [\mathrm{k}\mathrm{N}/{\mathrm{m}}^{2}]$	7
${m}_{K} [-]$	0.7	$s [-]$	1.1	$\psi [^\circ ]$	0
${a}_{0} [-]$	0.181E-3

**Table 2:** Coefficients of earth pressure for the soil layers

Soil layer	WLC	ILC	LGC
Coefficient of earth pressure (${K}_{0}$)	1.2	1.5	1.0

In all analyses, whether monthly or daily rates were applied, analysis results, including stress distributions which are needed for the calculation of FoS in secondary analyses, were saved only at the end of the month. This was done for practical purposes in this decades-long analysis (i.e. storing a total of 360 steps rather than 10,950). In any case, direct comparison with analyses where monthly rates were applied was only meaningful at the end of each month. As a result, transient reductions in stability during short-duration events, such as storms, were not explicitly studied. Their overall impact, however, was still present in the analysis and influenced the FoS computed at the end of the month.

### Hydraulic Soil Properties

The initial groundwater table was assumed at a depth of 1 m (Vaughan 1994). A Van-Genuchten Soil Water Retention Curve (SWRC) (Van Genuchten 1980) based on collected field data by Smethurst et al. (Smethurst et al. 2012) was used for the WLC, which was allowed to desaturate as suction increased, and its parameters are shown in Table 3. Also, a variable permeability model was used for the WLC layer, which decreases permeability with the degree of saturation as expressed below (Mualem 1976):

1 $${S}_{eff}=\frac{S-{S}_{res}}{{S}_{sat}-{S}_{res}}$$

2 $${k}_{rel}={\left({S}_{eff}\right)}^{{g}_{l}}.{\left\{1-{\left[1-{\left({S}_{eff}\right)}^{\frac{{n}}{{n}-1}}\right]}^{\frac{{n}-1}{{n}}}\right\}}^{2}$$

where ${S}_{eff}$, ${S}$, ${S}_{sat}$ and ${S}_{res}$ are the effective, current, saturated and residual saturation and n is a fitting parameter (the same as the Van-Genuchten parameter, presented in Table 3). ${g}_{l}$ is a fitting parameter, assumed to be 0.5 here to reproduce the initial expression by Mualem (1976). ${k}_{rel}$ is the relative permeability, which varied between 1 and 0.0001. The lower limit is imposed by PLAXIS to prevent the actual permeability from reaching 0 and causing numerical non-convergence.

**Table 3:** Van-Genuchten SWRC parameters

Parameter	$m$[-]	$n$[-]	$\alpha$[1/m]	${S}_{res}$[-]	${S}_{sat}$[-]
Value	1/3	1.5	0.15	0	1

The saturated hydraulic conductivity for the different layers is presented in Table 4 and is in accordance with Smethurst et al.’s data. Permeability for all soil layers was allowed to decrease with increasing mean effective stress using a User Defined Flow Model (UDFM) (Taborda, Tsiampousi, et al., 2023).

3 $${k}_{v}={k}_{y,ref}{e}^{a{p}^{^{\prime}}}$$

where ${k}_{y,ref}$ is the reference permeability along the y direction, ${k}_{v}$ is the current vertical permeability, ${p}^{^{\prime}}$ is the mean effective stress and $a$ is a fitting parameter. $a$ was taken as 0.007 $[{\mathrm{m}}^{2}/\mathrm{k}\mathrm{N}]$ in accordance with Kovacevic et al. (Kovacevic et al. 2007). The ratio of horizontal to vertical permeabilities for all soil layers, used to simulate the 2D nature of water flow, was chosen as 10, as proposed by Wongsaroj et al. (Wongsaroj et al. 2013).

**Table 4:** Hydraulic conductivity for the soil layers

Soil layer	WLC	ILC	LGC
Saturated hydraulic conductivity (${k}_{sat} [m/day]$)	3.715E-3	3.715E-4	3.715E-4

### Modelling Soil–Plant–Atmosphere Interaction (SPAI)

To model SPAI, a physics-based ecohydrological model, Tethys-Chloris (T&C), is used, which resolves coupled water, carbon and energy budgets at the soil surface in an hourly time scale (Fatichi 2021; Fatichi et al. 2012). This coupling allows vegetation to dynamically respond to weather forcings, which, in turn, affects soil-atmosphere interactions. T&C considers local weather data such as precipitation, short and long wave radiation, temperature, wind speed at 10 m, vapour pressure deficit, and ${CO}_{2}$ concentration, along with the soil’s hydraulic properties and SWRC, and calculates water infiltration, actual evaporation from bare ground, evaporation from intercepted water by plants, actual transpiration, and runoff. Maddah Sadatieh et al. (2025) demonstrated that it can calculate the net water inflow/outflow to be applied as a surface infiltration boundary condition in PLAXIS, yielding satisfactory agreement with measured PWP values (Maddah Sadatieh et al. 2025). The PLAXIS infiltration boundary condition is a dual boundary condition capable of automatically switching between prescribed pore pressure heads and an inflow/outflow water flux with user-defined rates. The maximum allowable head was set to -0.5 m (5 kPa of suction) to maintain a realistic water table. The minimum allowable head was set to -100 m (i.e., a suction of 1000 kPa) (Nyambayo & Potts 2010).

Table 5 presents the analyses conducted and the considerations made with respect to the temporal and spatial variation of the SPAI BCs, which are further explored in Sects. 3, 4, 5.

**Table 5:** Summary of the different numerical analyses considered

Analysis ID	Flux rates	Vegetation	Location of transpiration
Analysis A	Daily	Dynamic grass and shrubs	Surface
Analysis B	Monthly	Dynamic grass and shrubs	Surface
Analysis C	Daily	Static grass and shrubs	Surface
Analysis D	Daily	Dynamic tree	Surface
Analysis E	Daily	Dynamic tree	Distributed with depth

#### Past, Present and Future Weather Conditions

**Fig. 2:**
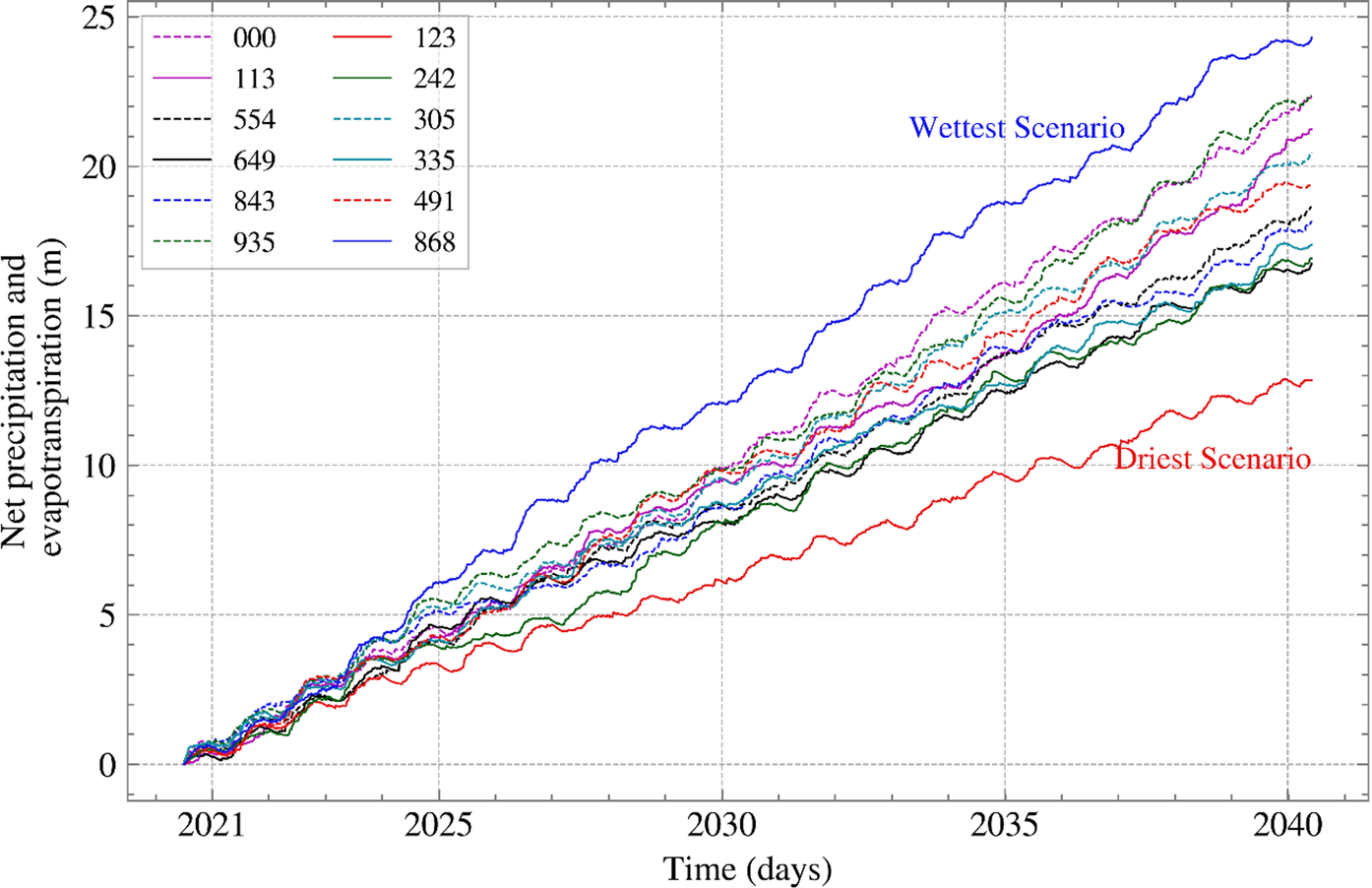
Net precipitation and evapotranspiration for UKCP18—RCP8.5 ensemble members to identify the extreme weather scenarios

The analyses were first run for the period covering the slope excavation and monitoring (i.e., July 1997 to December 2008), followed by a climate projection scenario from 2021 to 2040. For the climate projection, UKCP18 for the Representative Concentration Path 8.5 (RCP8.5) was used, which is the worst-case scenario where no climate mitigation is employed (Meinshausen et al. 2011; RCP Scenario Data Group 2024; UKCP 2024; UKCP18 Convection-Permitting Model Projections for the UK at 2.^2^ km Resolution 2024). After calculating the net inflow/outflow for the 12 ensemble members of UKCP18 (shown in Figure 2), the driest and wettest scenarios were identified. Having considered these two extreme scenarios in numerical analysis of the same slope, Maddah Sadatieh et al. (2025) demonstrated that the driest scenario was characterised by more pronounced seasonal variations of the computed pore water pressure and FoS. Its seasonal and interannual variability exceeding those of the wettest scenario and by extension of the scenarios in between, the driest scenario provides a means of capturing the expected range of variation with a single scenario, without the need of considering all ensemble members, that would be computationally unfeasible. The selected scenario, therefore, provides a plausible, high impact case for analysing the slope’s behaviour.

#### Vegetation

Three vegetation covers were assumed here. Smethurst et al. (2012) reported that the Newbury slope was covered by grass and shrubs, with a maximum root depth of 800 mm while monitoring took place (Smethurst et al. 2012). It was assumed here that shrubs covered 70% of the ground surface and grass the remaining 30%, with maximum root depths of 800 mm and 150 mm, respectively. Their Leaf Area Index (LAI) was calculated dynamically by T&C to respond to weather forcing and environmental stresses. This vegetation cover was used in Analyses A and B. In Analysis C, a static grass and shrubs vegetation cover was assumed, with the same root depths as described above, but with a constant LAI during the climate projection years, which was equal to the average LAI calculated in the previous scenario. In Analyses D and E, a tree cover with a maximum root depth of 2 m and dynamic LAI was considered.

**Fig. 3:**
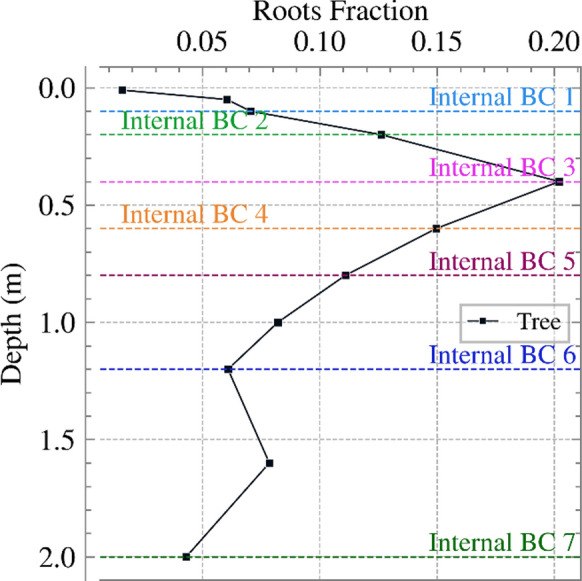
Root fraction variation against depth in the root zone for the tree cover

Distribution of fine roots within the soil can be defined in T&C by a parameter named “fraction of fine roots in soil layers”. This parameter represents the percentage of active water-absorbing fine roots at different soil depths. Here, it was assumed that the root distribution declines exponentially with depth. Figure 3 shows the root fraction function for the tree cover. In this approach, most of the root density was in the upper part of the root zone, near the ground surface.

#### Hydraulic Boundary Conditions

The calculated net inflow/outflow from T&C (as mentioned in Sect. 2.4) for each analysis was implemented as a surface BC in PLAXIS using the infiltration BC, as explained above. To reduce the computational cost of the geotechnical analysis, the hourly rates calculated from T&C were aggregated to a daily scale, with the exception of Analysis B, where monthly rates were applied, as detailed in Sect. 3. Analysis E is also an exception in that actual transpiration was not considered within the surface net inflow/outflow, but it was distributed with depth according to the root fraction in Figure 3. This is further explained in Sect. 5.

**Fig. 4:**
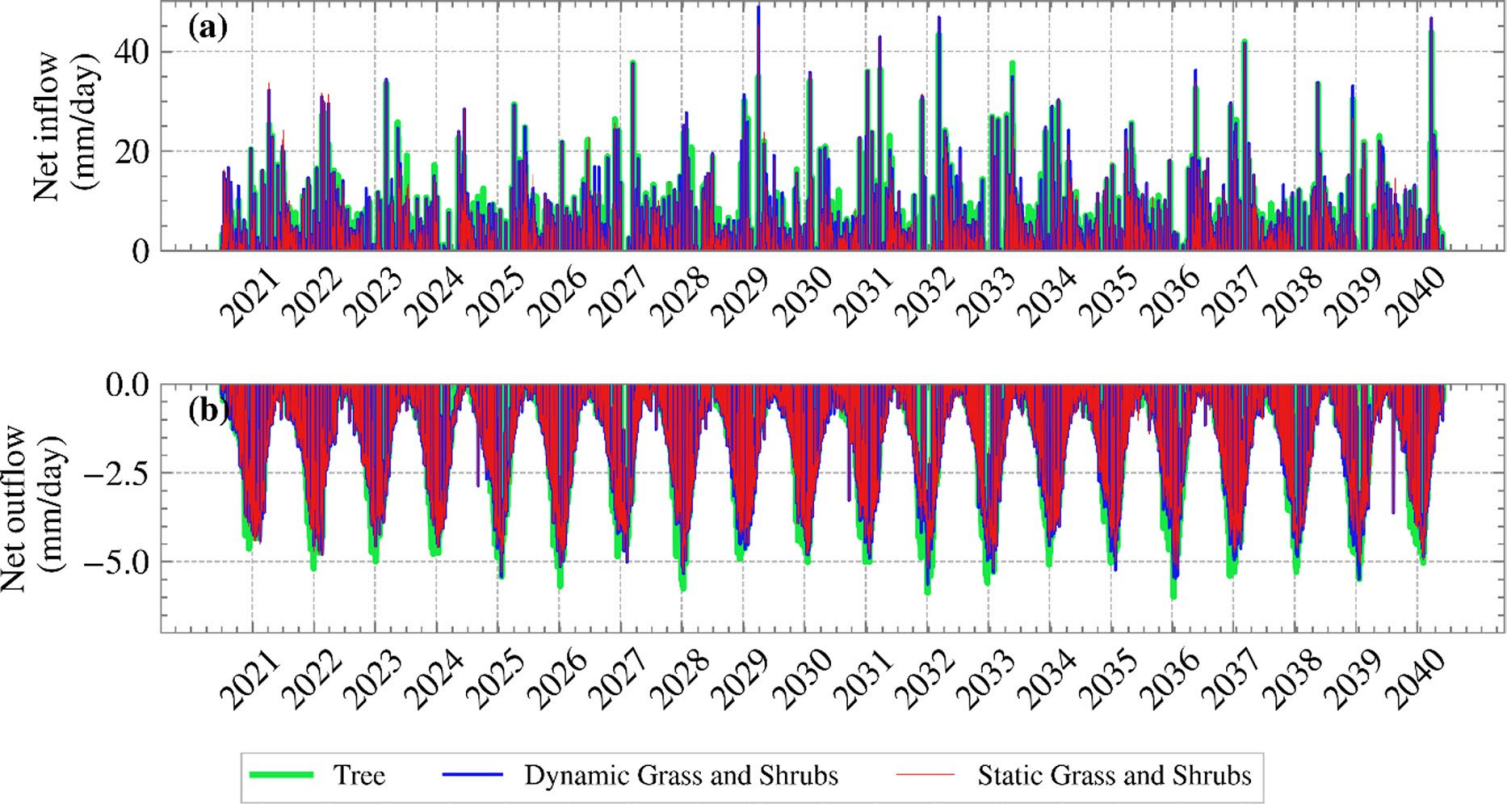
Net inflow/outflow against time for the years 2021 to 2040 for the three vegetation covers: dynamic grass and shrubs, static grass and shrubs, and tree cover

The axis of symmetry and the right vertical boundaries were impermeable, whereas water could move freely through the bottom boundary at the interface with the permeable chalk. The PWPs within the soil were allowed to change seasonally in accordance with the applied infiltration BC. After calculating net inflow/outflow for all three vegetation covers, they are shown for the climate projection years in Figure 4.

## Daily vs. Monthly Rates

**Fig. 5:**
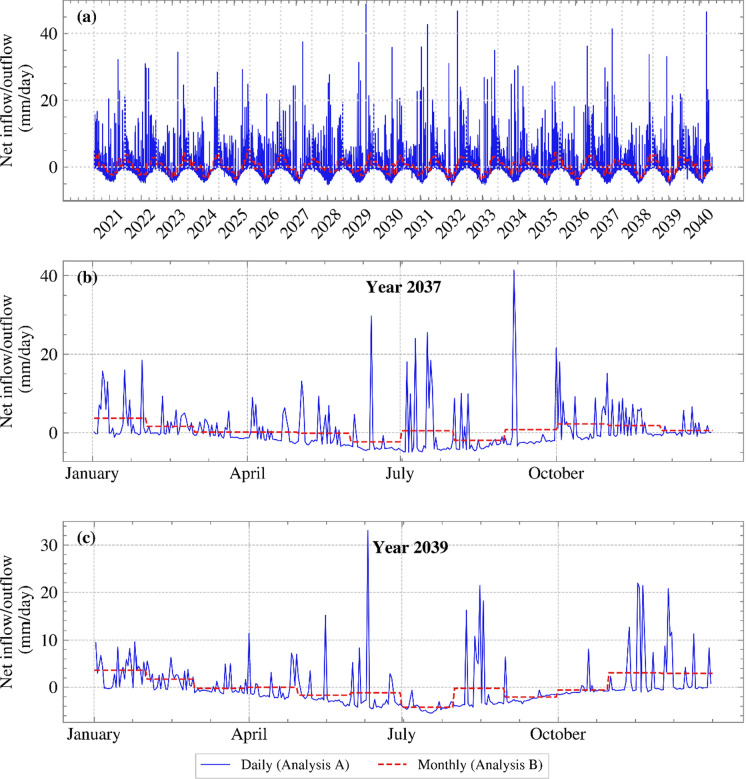
Daily and monthly net inflow/outflow against time for **a** the years 2021 to 2040, **b** the year 2037, and **c** the year 2039

As stated above, the hourly net inflow/outflow from T&C was aggregated into daily rates in the geotechnical model (Analysis A). As highlighted in the Introduction, SPAI BCs have been previously modelled on a monthly basis. To show the sensitivity of computed FoS and displacements to the temporal resolution of climatic inputs (i.e., the temporal resolution of the SPAI BC applied), the analysis was re-run with aggregated monthly rates (Analysis B) and compared with the daily rate analysis (Analysis A). Figure 5 (a) compares the applied daily and monthly BCs. Evidently, aggregating weather date on a monthly basis, averages out daily extremes. This is shown clearly in Figure 5 (b) and (c) which zoom in for years 2037 and 2039, respectively.

**Fig. 6:**
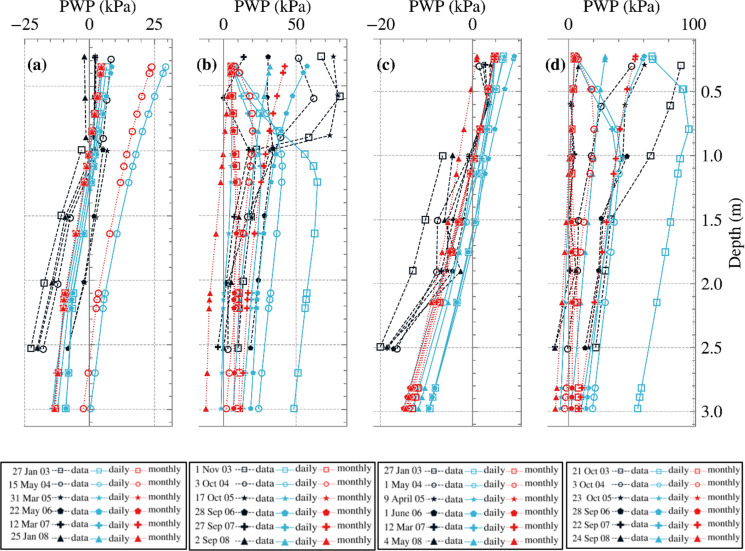
Comparison of PWP variations with depth for Analyses A and B and field data: **a** winter and **b** summer months for location A, and **c** winter and **d** summer months for location C (suction is shown as positive)

Analysis A had been validated by Maddah Sadatieh et al. (2025) by introducing the methodology used here by integrating a physics-based ecohydrological model and coupling that with a geotechnical software and comparing the computed PWP with measured values reported by Smethurst et al. (2012) at locations A and C (see Figure 1) (Maddah Sadatieh et al. 2025; Smethurst et al. 2012). Figure 6 illustrates the variations in PWP with depth at the same locations for both Analyses A (daily) and B (monthly) and field data. The symbols along the dashed black line indicate PWP measurements, with the dashed line in between representing the interpolation between the measured data points. The symbols on the blue and red lines show the spatial resolution of the model. Tensile PWP (i.e., suction) is plotted as positive throughout this study. The Mean Absolute Error (MAE) and Root Mean Squared Error (RMSE), summarised in Table 6, indicate that errors were lower in the winter months than in the summer months, and that the daily analysis outperformed the monthly in the winter but not in the summer. Considering the uncertainties in both the field measurements and the numerical analysis, these errors are generally acceptable. Although Analysis B produced PWPs within the measured range, there were fewer distinctions between different months, seasons, and even years, whereas these differences were more pronounced in Analysis A. Having smoothed out net inflow/outflow extremes, the coarser monthly time step in Analysis B also smoothed out differences in PWP profiles. Applying seasonal or even monthly BCs omits the timing and occurrence of individual storm or drought events, whereas a daily transient simulation is better able to capture day-to-day changes. For example, long draughts and short, intense rainfall events can cause surges in tensile and compressive PWP, which can be captured under fine temporal resolution in BCs but are lost when data are aggregated monthly.

**Table 6:** Quantitative comparison of PWP variations for Analyses A and B and field data

Season	Location	$\tt {MAE}_{daily}$[kPa]	$\tt {RMSE}_{daily}$[kPa]	$\tt {MAE}_{monthly}$[kPa]	$\tt {RMSE}_{monthly}$[kPa]
Winter	A	6.163	8.322	6.282	8.377
Summer	A	25.646	30.412	10.524	12.994
Winter	C	4.696	5.801	5.910	7.260
Summer	C	30.236	39.548	8.117	13.570

**Fig. 7:**
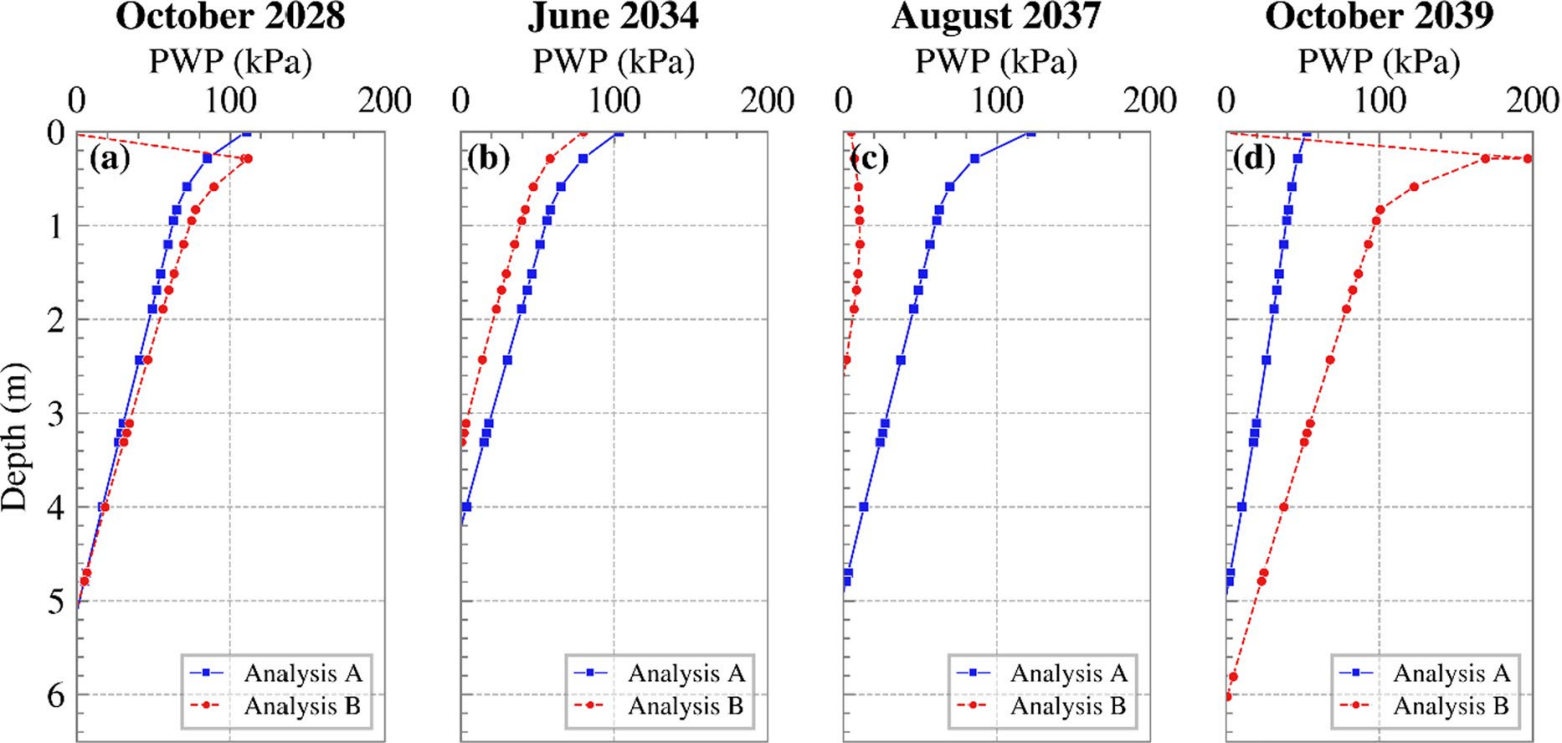
Cross sections of changes of PWP vs. depth at a few instances for Analyses A and B at mid-slope (suction is shown as positive)

Figure 7 illustrates the distribution of PWP with depth mid-slope, at selected times. These profiles allow a direct comparison of the effect of temporal resolution on soil suction responses, demonstrating how the aggregation of climatic inputs influences the calculated suction distribution throughout the top few metres. Although there are instances and depths where the two analyses yield similar suctions, at times one analysis predicts higher suctions than the other, however, without a clear, consistent or identifiable trend. These differences can extend to depths of approximately 6 m from the soil surface, therefore potentially affecting slope behaviour.

**Fig. 8:**
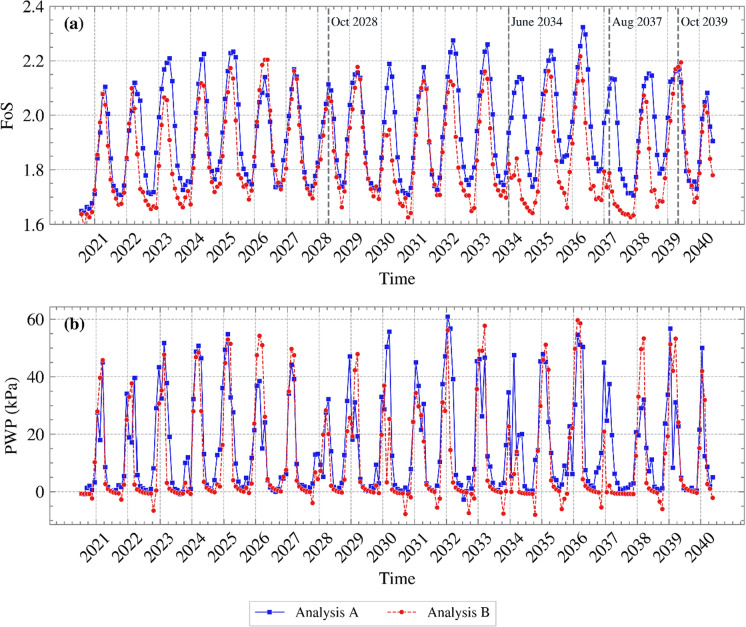
**a** FoS variation with time and **b** PWP variation with time near the slope’s toe, for the years 2021 to 2040 for Analyses A and B (suction is shown as positive)

**Fig. 9:**
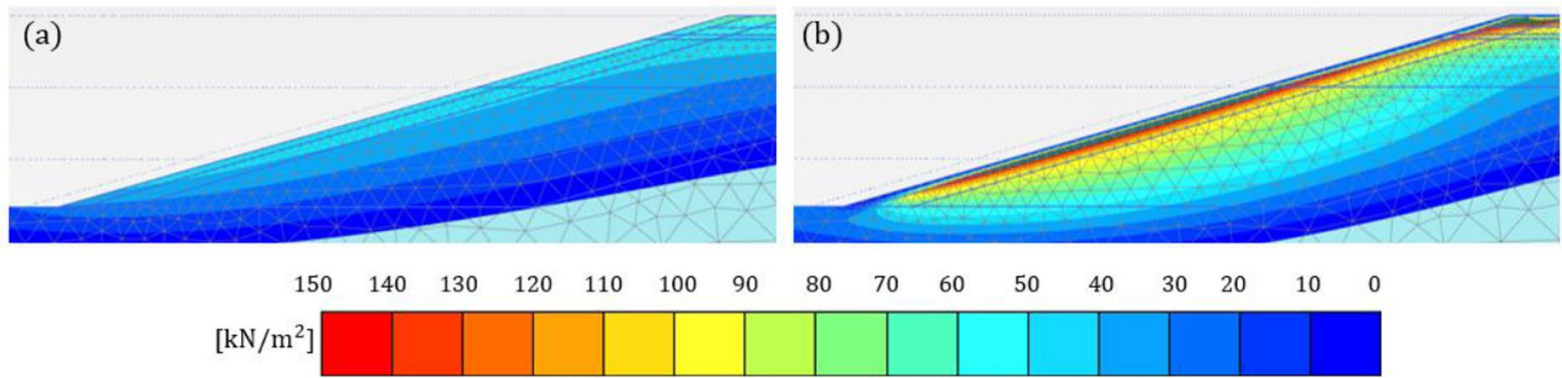
Suction contours in October 2039 for **a** analysis A and **b** analysis B (suction is shown as positive)

To examine the potential impact on stability, FoS variations over time are plotted in Figure 8 (a) for each month from 2021 to 2040. Figure 8 (b) shows the PWP time series near the slope’s toe (0.2 m in depth). Generally, Analysis B yielded lower FoSs in both summer and winter, with a few exceptions. This is consistent with the PWP profiles shown in Figure 7. Similar PWP profiles in October 2028 yielded a similar FoS; in June 2034 Analysis A produced slightly higher PWPs and FoS; the difference in PWPs and FoS between the two analyses was larger in August 2037. Interestingly, the difference in PWP in October 2039 did not translate to a measurable difference in FoS. Examination of the corresponding suction contours (shown in Figure 9) indicates that in Analysis A higher suctions were maintained around the slope’s toe, where slope failure mechanisms are typically initiated from when interacting with the atmosphere (Tsiampousi et al. 2017), in comparison to Analysis B. The higher suctions at the toe compensated for the lower suctions further up the slope, and the two analyses produced comparable values of FoS. This was probably coincidental, but it suggests that, although PWPs are commonly evaluated along cross sections in standard field monitoring practices, the global slope stability and behaviour may be disguised and not fully captured by such localised assessments.

Analysis B underestimated the FoS of Analysis A in years with many, isolated rainfall events, such as 2037, but provided a reasonable estimate in other years, such as in 2039. To examine this further, the daily and monthly SPAI BCs applied for the years 2037 and 2039 have been replotted in Figure 5 (b) and (c), respectively. It can be seen that the year 2037 is characterised by many, large daily spikes and variations, which were smoothed out by the average monthly rates in such a way that, for this particular year, most of the months show a monthly inflow (positive net flow), failing to capture water removal adequately in between rainfall events, and resulting in lower predicted FoS. In contrast, in 2039, the monthly rates averaged daily variations in a way that produced both net outflows and net inflows. This difference in the two example years occurred despite the fact that the cumulative net inflow/outflow for the monthly and daily rates was the same in each analysis and is due to the arbitrariness of the time step (monthly, as well as daily, time steps are a human convention). Another important note is that, depending on the saturation and operational permeability of the soil, concentrated intense rainfall events may not translate necessarily to actual water infiltration when applied as a daily BC, but may result in runoff. However, rainfall events are averaged and applied during a more extended period of time, such as a month, the actual water infiltrating the soil could be greater.

Analysis B could be considered conservative in terms of average FoS values. This is due to the smoothing of intense drying/wetting cycles (e.g., years 2034 and 2037), which reduces the short-term extremes and delays the recovery of suction. Therefore, monthly steps could potentially be used for studies that simulate decades, without a significant compromise in the stability calculation of the slope. When studying the effect of specific storms on the FoS, monthly steps could be used to initialise the analysis (e.g., from construction to the present day), so as to start the simulation of the storm from a representative PWP state, and adopt daily steps in the months preceding the storm event, reducing the time step further during the event to capture it accurately.

It should be acknowledged that conservatism in FoS may lead to overdesign or to unnecessary remedial measures. As the slope demonstrated healthy FoS values throughout, this would not have been a concern here.

**Fig. 10:**
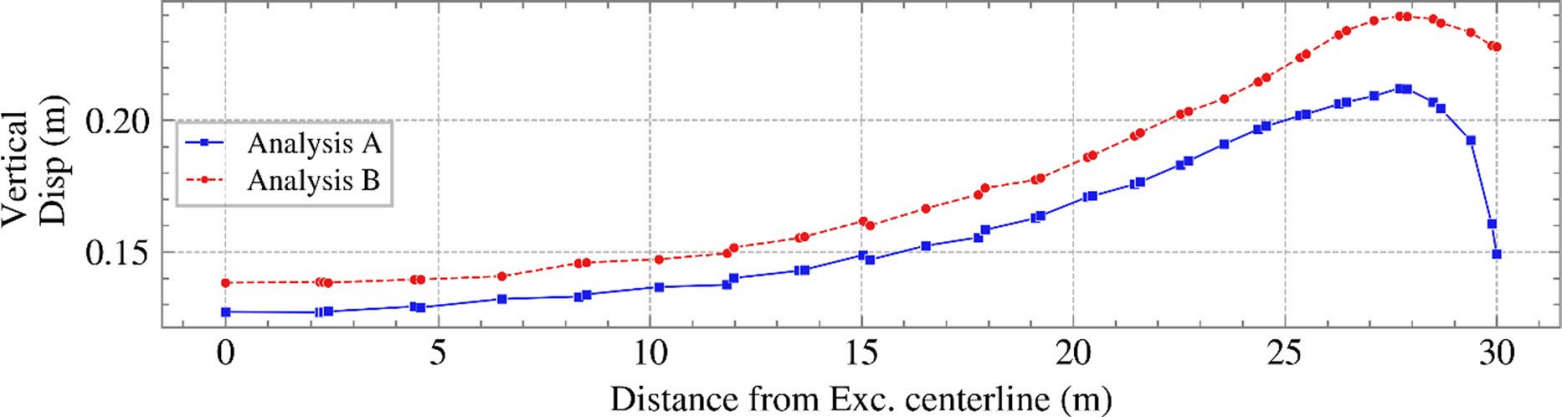
Vertical displacement at the bottom of the excavation against distance from the excavation centerline for Analyses A and B at the end of 2040

**Fig. 11:**
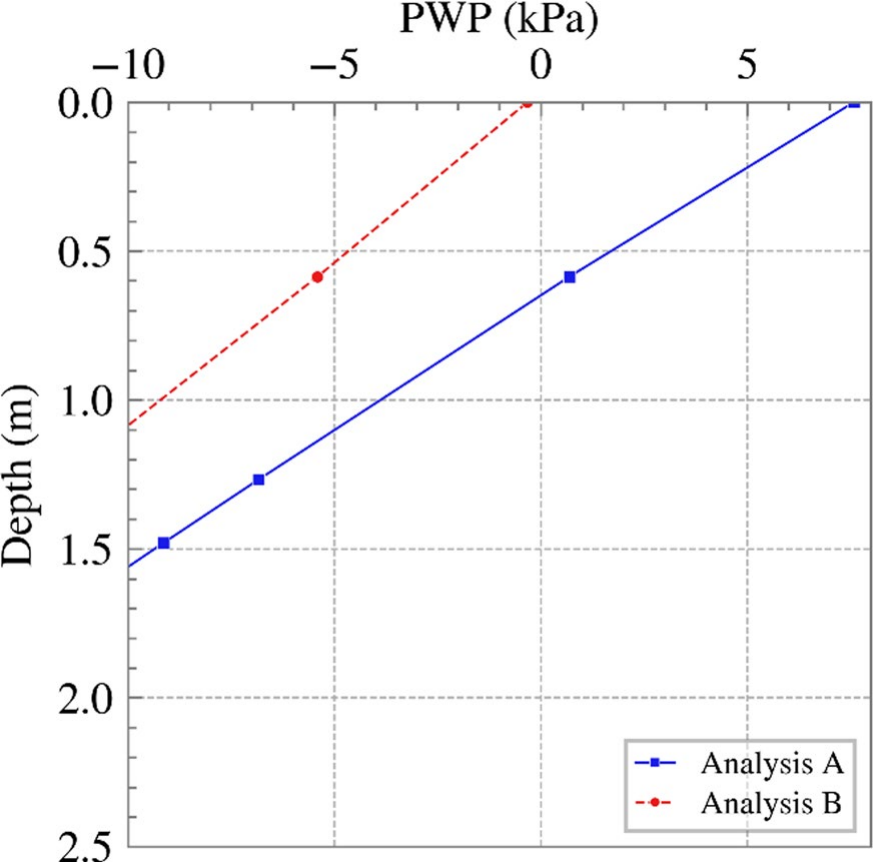
Variations of PWP vs. depth at the end of the analysis (November 2040) at the toe of the slope (suction is shown as positive)

To examine the impact of time step on serviceability, vertical displacements at the bottom of the excavation were considered. Figure 10 shows the vertical displacement against the distance from the excavation centerline (i.e., the road shown in Figure 1) at the end of the analysis (i.e., year 2040 – the end of the climate projection). In addition to the differences in the actual vertical displacement in the two analyses, the differential displacements were markedly different near the toe of the slope, where more shrinkage was observed in Analysis A. This was close to where SPAI was applied, and, therefore, it can be observed that implementing it as daily or monthly rates may have a significant impact. Considering only the distance from the maximum displacement to the centerline and neglecting the area around the toe, the differential displacement was larger in Analysis B, again providing conservatism, but potentially leading to unnecessary maintenance practices. The PWP profiles with depth in November 2040 at the toe of the slope are shown in Figure 11. Whereas pore water pressures were compressive in analysis B, suctions were retained in Analysis A, which resulted in the shrinking observed in Figure 10 (or potentially smaller swelling in comparison to Analysis B). This is explained by the daily/monthly rates seen in Figure 5 (a), where net inflow was applied in the last months of Analysis B, while the daily rates in Analysis A included net outflows, which aided some small suctions to be retained.

## Dynamic vs. Static Vegetation

**Fig. 12:**
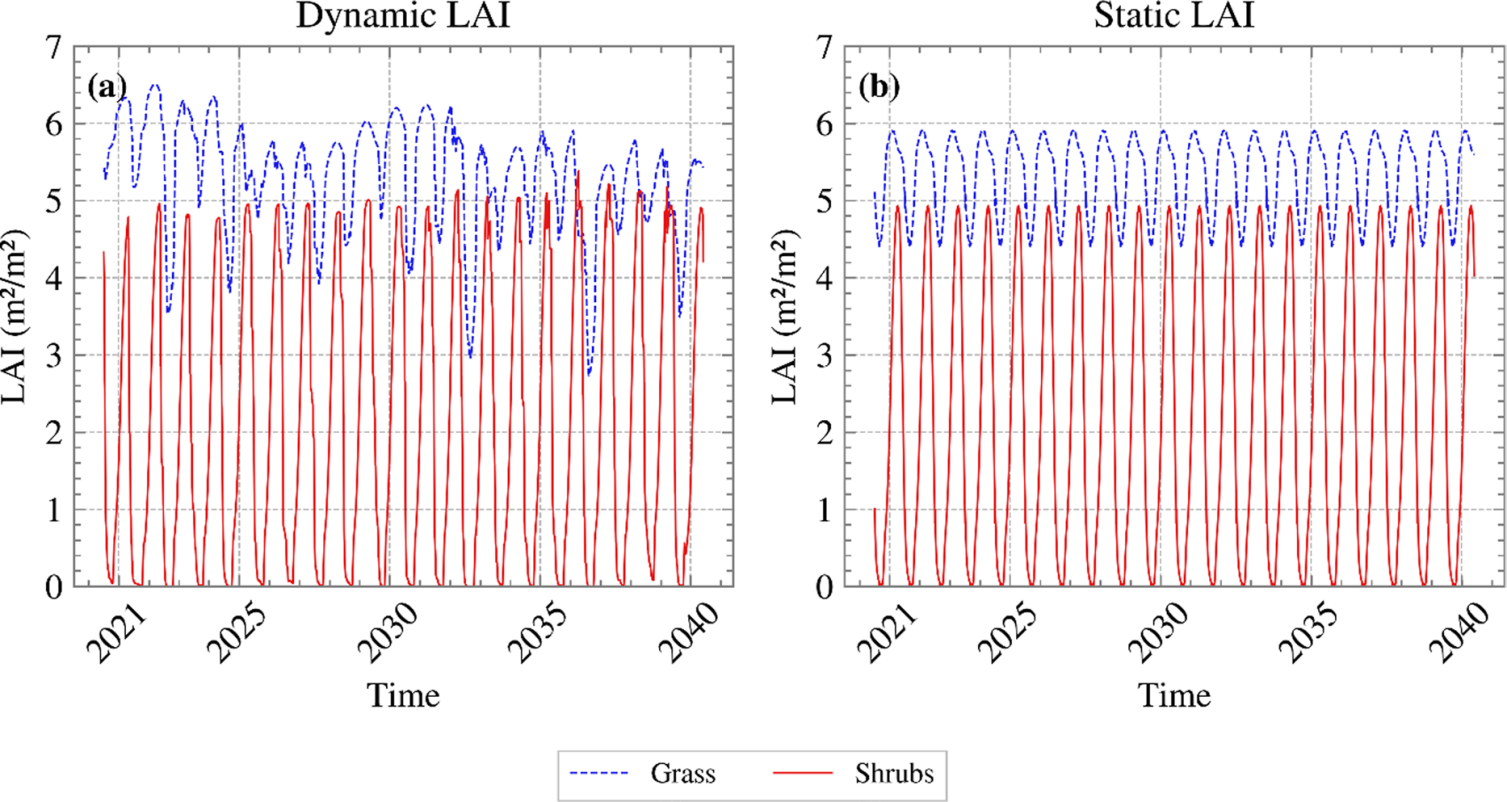
LAI for **a** dynamic and **b** static vegetation covers

To take a closer look at the effect of modelling vegetation dynamically or statically, two analyses were run: Analysis A, in which LAI evolved in response to environmental conditions, and Analysis C, in which an average annual LAI variation was applied. The average annual LAI was calculated as a daily arithmetic average of the 20 years of dynamic vegetation simulation. Although LAI in Analysis C did not change year to year, it still reflected seasonal trends. Since it was derived from 20 years of dynamic vegetation, it captured the general trends observed across the full period; this reduced the need to tune parameters to specific vegetation and calibrate all inputs, rendering calibration of the ecohydrological model more straightforward and less time-consuming. Figure 12 depicts the LAI for both dynamic and static vegetation covers.

**Fig. 13:**
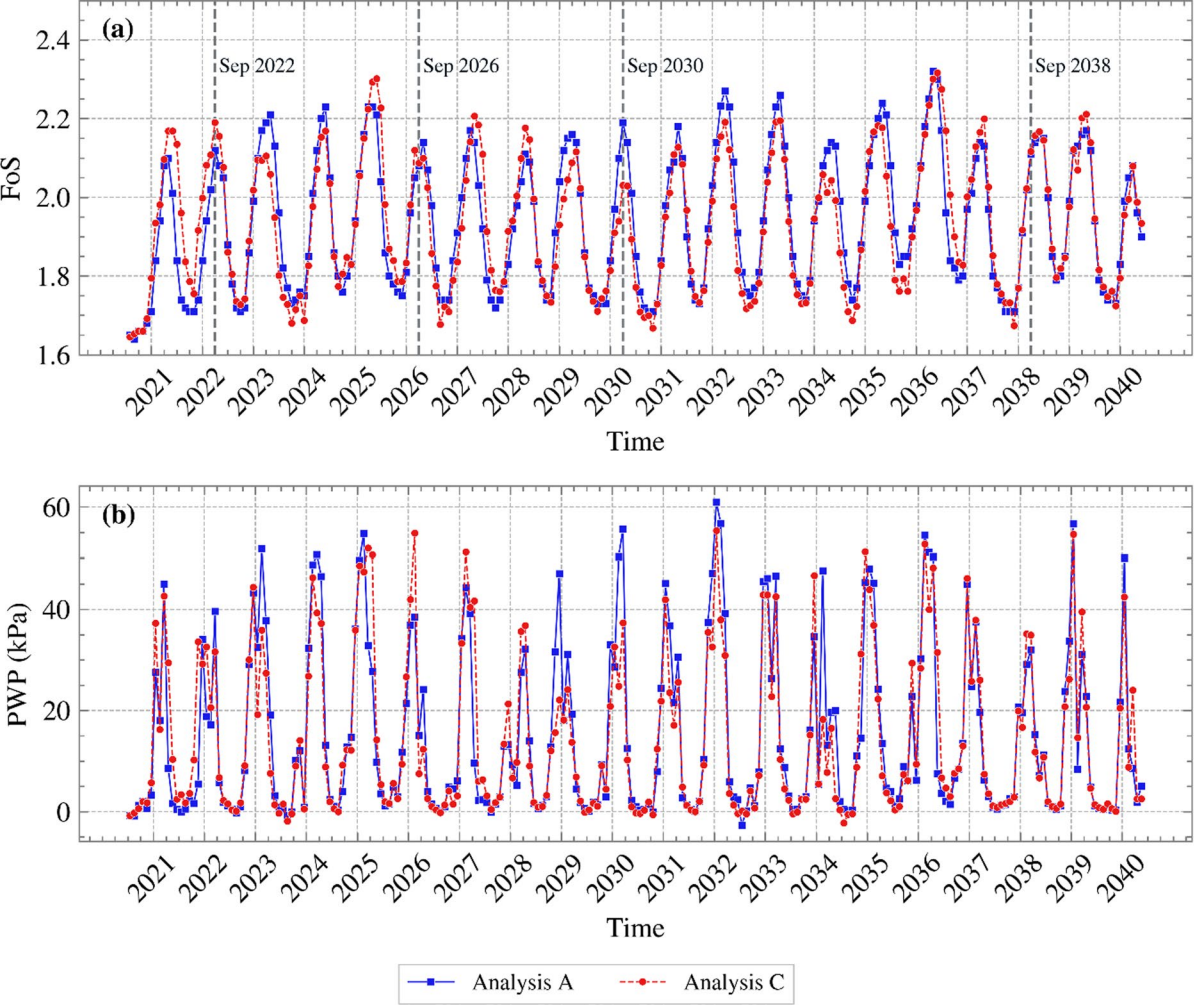
**a** FoS variation with time and **b** PWP variation with time near the slope’s toe, for the years 2021 to 2040, for both Analyses A and C (suction is shown as positive)

Figure 13 (a) depicts the variation of FoS with time for the years 2021 to 2040 for Analyses A and C. Figure 13 (b) shows the PWP time series near the slope’s toe (0.2 m in depth). FoS for the two cases were quite similar, with no clear trend of one overpredicting the other. In general, simulating dynamic vegetation demonstrated only small, negligible differences in FoS in relation to static vegetation. This indicates that uncertainties in vegetation parameters affecting LAI may be safely disregarded when it comes to assessing slope safety. This is in contrast with Chen et al. (2025) who, assuming a constant value of LAI for static vegetation, i.e., disregarding inter annual variation, concluded that vegetation dynamics influence the magnitude of floods and landslides. Here, capturing the seasonal annual variations and averaging dynamic vegetation growth resulted in an acceptable representation of dynamic vegetation. This simplified the numerical model, while maintaining the accuracy that stems from T&C calculating all parameters at an hourly timescale.

**Fig. 14:**
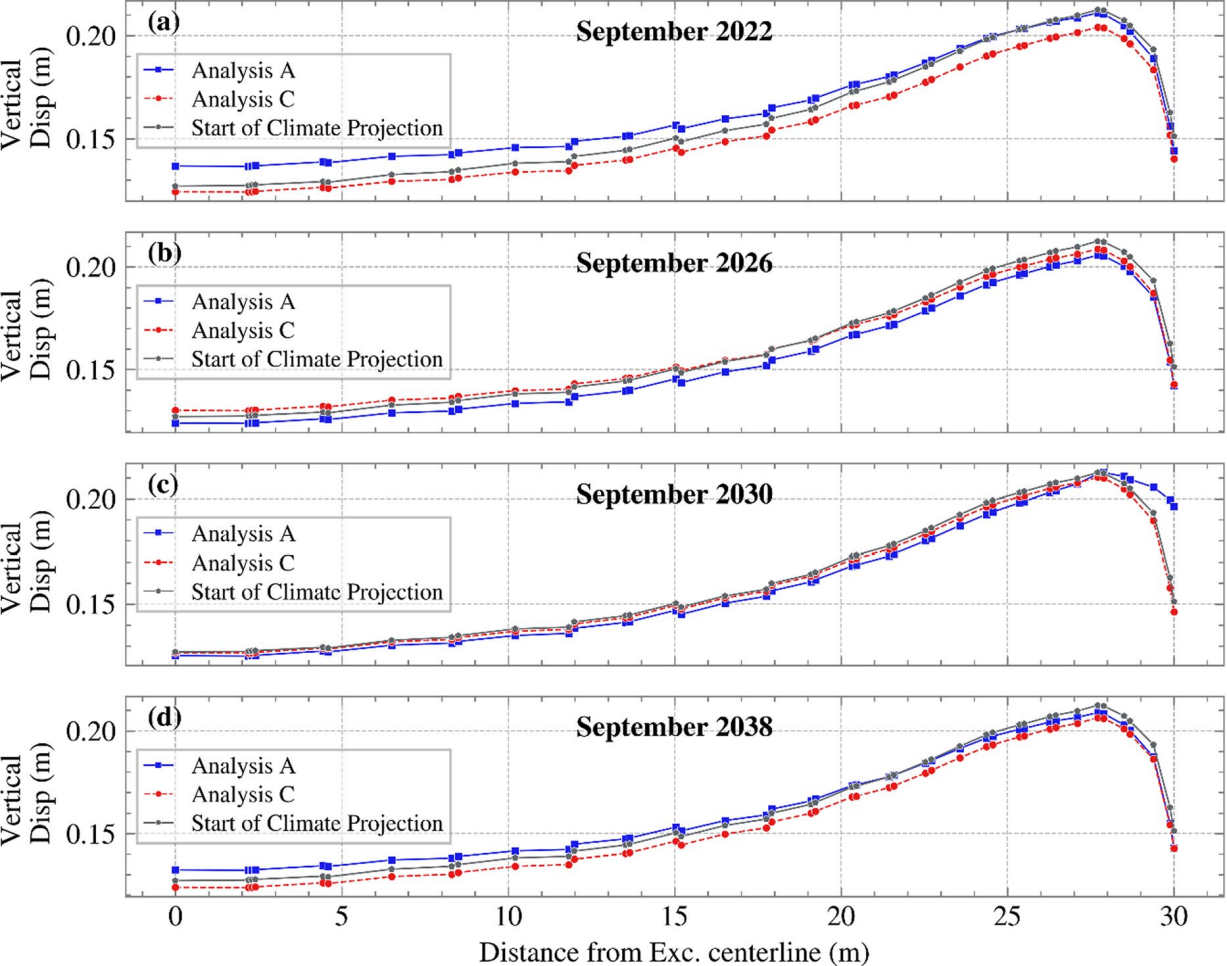
Vertical displacement against distance from the excavation centerline until the slope’s toe for months **a** September 2022, **b** September 2026, **c** September 2030 and **d** September 2038 for both Analyses A and C

Figure 14 illustrates the vertical displacement at different time instances for analyses A and C, relative to the distance from the excavation centerline to the slope toe. Results indicated minimal differences in vertical displacement and maximum differential displacement for the two vegetation covers, with few exceptions (e.g., in September 2030 when Analysis A showed less shrinkage near the slope’s toe). It is often assumed that vegetation and its water extraction negatively influence serviceability. It was demonstrated here that modelling vegetation with dynamic or static LAI had a negligible impact on the computed serviceability. In almost all cases, both the magnitude of vertical displacement and, most importantly, the maximum differential displacement were similar.

## Single Surface BC vs. Surface and Multiple Internal BCs

To investigate the effect of distributing water extraction due to transpiration with depth, Analyses D and E were conducted using tree cover with a deeper root zone. Analysis D used a surface BC where the net inflow/outflow was applied at the surface, as in previous cases. For Analysis E, transpiration was separated from the calculated net inflow/outflow applied to the surface. Specifically, precipitation, evaporation from bare ground and snow, intercepted evaporation and runoff were applied to the soil surface using the infiltration BC in PLAXIS, while transpiration was represented within the root zone using the outflow BC in PLAXIS. The transpiration rate was distributed according to the root fraction shown in Figure 3. In T&C, the 2 m deep root zone was subdivided into 11 “sub-layers” and the root fraction for each sub-layer is shown in Figure 3 (as points). The amount of transpiration from each sub-layer was obtained by multiplying its root fraction by the total transpiration. This was then applied in the geotechnical model as seven internal outflow BCs at depths of 0.1, 0.2, 0.4, 0.6, 0.8, 1.2, and 2 m from the soil surface (Figure 3). The 11 sub-layers were aggregated into seven internal BCs to avoid generating a very fine mesh and thus increasing the computation cost of the geotechnical analysis. The transpiration rates calculated from T&C for sub-layers 1, 2, and 3 were summed and applied in the first internal BC, sub-layers 8 and 9 were applied in the sixth internal BC and sub-layers 10 and 11 were applied in the seventh internal BC. Aggregation was only applied to layers with root fractions less than half of the maximum root fraction value. Layers with higher root fractions were assigned directly to individual internal BCs to preserve accuracy where rooting effects are most significant.

**Fig. 15:**
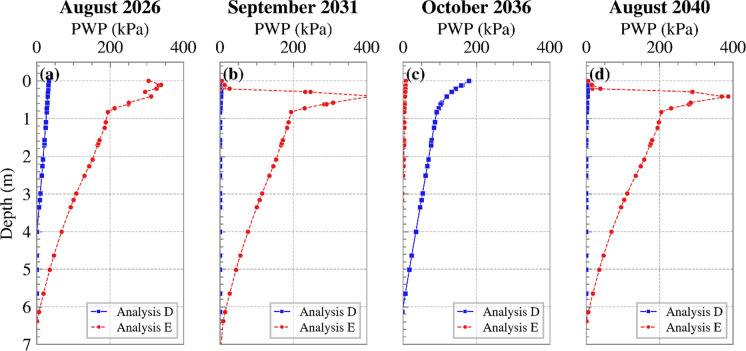
Cross sections of changes of PWP vs. depth at mid-slope in **a** August 2026, **b** September 2031, **c** October 2036 and **d** August 2040 for both Analyses D and E (suction is shown as positive)

Figure 15 shows the variations of PWP with depth for Analyses D and E at the time instances when the greatest differences in suction were exhibited. A consistent trend could not be seen for these two analyses, with instances where Analysis E yielded higher suctions and others with Analysis D having larger suctions. Modelling transpiration to occur with depth is expected to lead to larger suctions at the affected depths in the dry seasons. This is clearly observed in August 2026, September 2031 and August 2040. As wet period starts, the net inflow at the ground surface is smaller in Analysis D, as rainfall is offset by the whole of computed transpiration. This is in contrast with Analysis E where transpiration, albeit reduced, is distributed with depth. This explains the PWP profiles in October 2036. It is also worth noting that the effects of transpiration remained noticeable beyond the root zone, with suctions sustained at depths larger than 2 m, as suggested by Tsiampousi et al. (Tsiampousi et al. 2017).

**Fig. 16:**
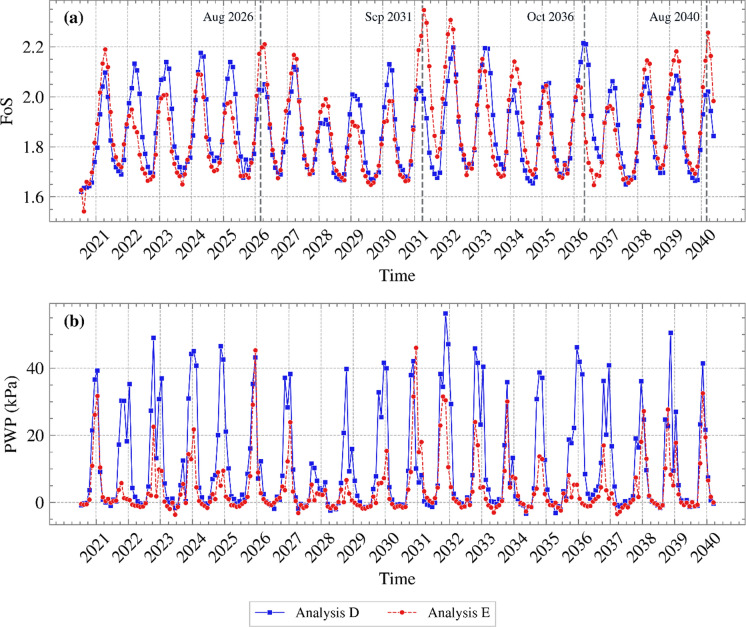
**a** FoS variation with time and **b** PWP variation with time near the slope’s toe, for the years 2021 to 2040, for Analyses D and E (suction is shown as positive)

FoS against time is illustrated in Figure 16 (a) and Figure 16 (b) shows the PWP time series near the slope’s toe (0.2 m in depth). In accordance with the respective PWP profiles shown in Figure 15, higher FoS values were associated with the analysis yielding higher suctions. The vast differences in suction in the two analyses resulted in differences in FoS that far exceeded the differences observed between previous analyses. The maximum difference of approximately 0.35 observed in FoS values in 2031 was perhaps immaterial for this well-designed slope; however, it would certainly be of significance in slopes where stability would be predicted to be marginal, with different analyses predicting different outcomes.

**Fig. 17:**
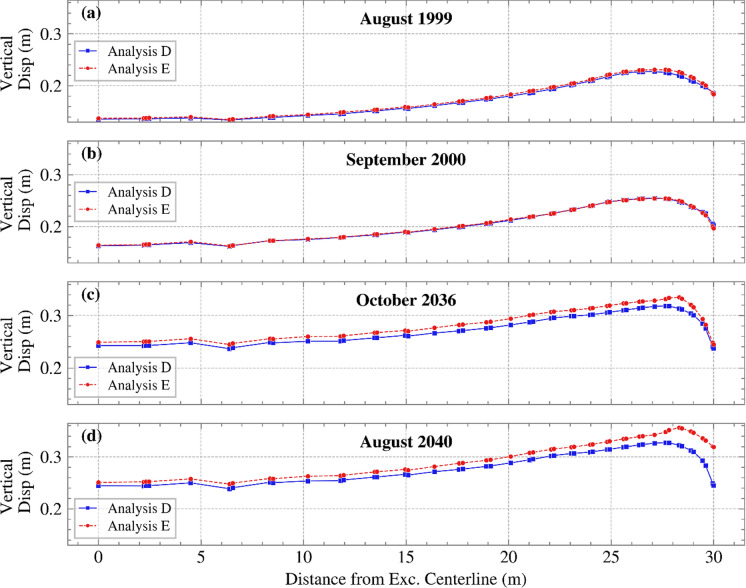
Vertical displacement against distance from the excavation centerline until the slope’s toe for months **a** August 1999, **b** September 2000, **c** October 2036, and **d** August 2040, for Analyses D and E

The vertical displacement against distance from the excavation centerline to the slope toe is plotted in Figure 17. This has been done for time instances when maximum differences in suction were observed, for both the initial years (July 1997 to 2008) and the climate projection period (2021 to 2040). Interestingly, in the initial years (Figure 17 (a) and (b)), the vertical displacements showed little to no difference between the two analyses, whereas in climate projection years (Figure 17 (c) and (d)), the differences between the analyses became visible. Maddah Sadatieh et al. (2025) has demonstrated how vertical displacements at the bottom of the excavation can be related to changes in suction between successive dry and wet seasons.

**Fig. 18:**
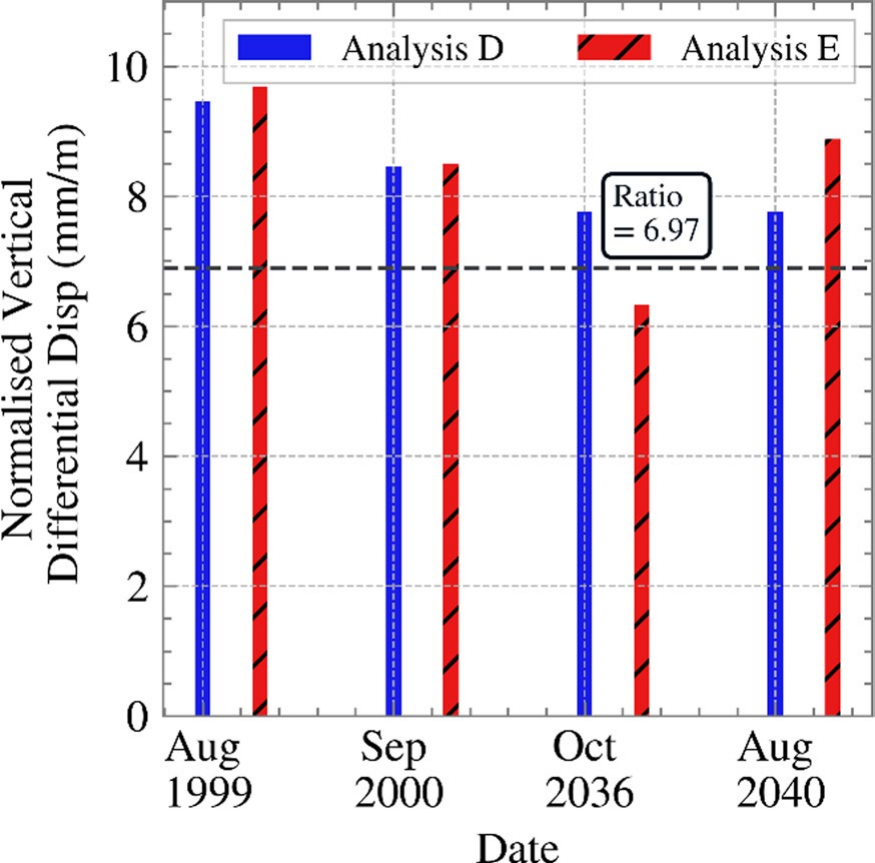
Normalised ratio of the vertical displacement for months showing larger differences in suction, for both Analyses D and E

The maximum differential displacement at the bottom of the excavation, which is key for serviceability, remained practically the same for these two analyses during the initial period. When climate change was taken into account in subsequent years, these differences became more visible over time. To put the differential displacements calculated into perspective, they are compared with the Network Rail’s cross-level maintenance tolerance of 10 mm (Network Rail 2022), which for a 1,435 mm track gauge, corresponds to a normalised ratio of 6.97 (10 mm over 1.435 m). Figure 18 illustrates the maximum normalised ratio for the months plotted in Figure 17. As demonstrated, the ratios at times exceeded the maintenance threshold set by Network Rail. Although the site refers to a road, this threshold remains indicative of potential disruption in geotechnical infrastructure in general.

## Summary and Conclusion

This paper examines different approaches to applying Soil–Plant-Atmosphere Interaction (SPAI) Boundary Conditions (BC) and their influence on Pore Water Pressure (PWP) distribution and displacements, which ultimately influence safety and serviceability. The general methodology from the authors’ previous work, whereby an ecohydrological model (T&C) was employed to compute net inflow/outflow BCs and apply them in PLAXIS, was modified to consider three distinct approaches: 1) daily vs. monthly rates, 2) dynamic vs. static vegetation, and 3) surface transpiration vs. transpiration distributed with depth. In order to investigate how the application of BCs impacts slope behaviour in future years under climate change, 2D analyses of the slope were performed, and suction profiles, PWP time series near the slope's toe, monthly Factors of Safety (FoS), and vertical displacements were evaluated and compared. It was shown that:

- Daily vs. monthly rates: This aggregation demonstrably influences results. By applying monthly BCs, overall general trends were still captured, but there was little difference between different months and even years. The maximum differential displacements were also affected. Monthly rates generally produced conservative results both in terms of stability and serviceability. It is appreciated that conservatism in design may lead to unnecessary economic and environmental costs. Monthly time steps may be suitable for studies simulating periods spanning several decades. For analyses examining the impact of specific storm events on stability and serviceability, monthly steps can be employed to initialise the model from construction to present day, ensuring a representative PWP state, before the event. Subsequently, daily time steps should be utilised in the months leading up to the storm event, with further time step reductions during the event itself to enable accurate simulation.
- Dynamic vs. static vegetation: If a reasonable average LAI is used, the static analysis can capture both safety and serviceability effectively. Although the dynamic vegetation’s LAI responded more realistically to climate conditions and their changes, it required more data and time to calibrate the T&C model. Therefore, it is advantageous to use annually static, seasonably varying LAI, as this renders calibration of the ecohydrological model more efficient, without compromising the geotechnical output. This acceptable approximation can potentially enable the direct use of satellite gathered LAI data, which are usually available for different regions and vegetation types.
- Surface transpiration vs. transpiration distributed with depth: Suction changes with depth were examined, and it was shown that substantial variations in PWP distribution arose, subsequently affecting FoS and vertical displacements. A maximum difference in FoS of approximately 0.35 was computed here. This may be significant for slopes whose stability is critical, while it may be immaterial for well-designed slopes. The surface only BC model captured seasonal variations and overall magnitudes of suction effectively enough, providing to be sufficient for the analysed slope as it captured the necessary aspects for slope stability and serviceability in general. It should be emphasised that this conclusion should not be readily extended to all slope geometries and all vegetation covers. For instance, the output will likely depend on the depth of the root zone and the assumed root distribution. With particular reference to serviceability, differences between different approaches may become more pronounced under the influence of climate change.

These different approaches may be selected depending on the goal of the analysis and can be used to provide guidance for practitioners. Table 7 summarizes the recommended modelling approach for different project stages.

**Table 7:** Summary of guidance for selecting different modelling approaches

Project stage	Accuracy	Modelling simplification
Preliminary design	Moderate	Static, surface, and monthly BC
Final design	High	Dynamic, surface, and monthly BC
Early warning	High	Dynamic, internal, and daily BC
Back analysis	High	Dynamic, internal, and daily BC

It is worth noting that the mechanical contribution of vegetation cover was not considered in this study, which focused exclusively on the impact of hydraulic reinforcement through root water uptake and its modelling in numerical analysis. Although this limitation is expected to have had some quantitative impact on the computed factors of safety, their relative magnitudes and conclusions drawn in relation to hydraulic reinforcement are still valid, as in all comparisons the same type of vegetation was considered, nulling the comparative effect of mechanical reinforcement, while the FoS values remained conservative. Modelling of mechanical root reinforcement in numerical analysis is a wide and important topic, which merits its own study. Having quantified the effect of different considerations in relation to the hydraulic reinforcement, whose effects are far more difficult to anticipate by intuition, this study has laid the ground for research on the mechanical reinforcement under soil-atmosphere interaction both in isolation from the hydraulic reinforcement as well as concurrently with it, as it has provided a robust benchmark.

This study was conducted for a specific cut slope in London Clay which is a highly overconsolidated, stiff plastic clay and has low hydraulic conductivity. As such, the findings of this study are most applicable to engineered slopes in low-permeability, stiff clays, where seasonal pore-pressure cycles are driven primarily by atmospheric fluxes reflective of a temperate oceanic climate and shallow root water uptake. For soils with higher hydraulic conductivity, the relative importance of internal transpiration boundary conditions and transient flux variability may be amplified. Under different projected climate scenarios for the region, the general findings are expected to remain applicable. This was demonstrated previously by the authors (Maddah Sadatieh et al. 2025) who established similar overall trends in slope stability and serviceability across extreme climate scenarios. The conclusions regarding temporal resolution (daily vs. monthly rates) and dynamic versus static vegetation are directly relevant to slopes covered by grasses, shrubs, or moderately deep-rooted trees with root zones extending up to approximately 2 m, and potentially applicable to other vegetation covers, since the methodology relies on net inflow/outflow, which can be readily calculated for any vegetation type. However, the applicability of the findings for the surface and the internal BCs, depends on vegetation type, specifically their root zone depth. For deep rooted vegetation, internal BCs should extend deeper within the root zone, which is expected to result in larger differences in comparison to a surface BC. Therefore, for soils with significantly higher hydraulic conductivity, for deeper root systems, or for slopes subjected to substantially different climatic regimes (e.g., tropical) the trends identified here should ideally be re-evaluated with site-specific analyses following the methodology established in this paper. Although further research is required to generalise the findings of this study, the employed methodology can be applied to any problem. Furthermore, the conclusions drawn provide a solid basis on which to build further studies on soil–plant-atmosphere interaction and the use of geotechnical numerical analysis.

## Data Availability

The datasets generated during and/or analysed during the current study are available in the “vegetation parameter files” repository, 10.5281/zenodo.17826936.
